# Analysis of attempted suicide episodes presenting to the emergency department: comparison of young, middle aged and older people

**DOI:** 10.1186/s13033-020-00378-3

**Published:** 2020-06-22

**Authors:** Soo Hyun Kim, Han Joon Kim, Sang Hoon Oh, Kyungman Cha

**Affiliations:** 1 Department of Emergency Medicine, Eunpyeong St. Mary’s Hospital, College of Medicine, The Catholic University of Korea, Seoul, Republic of Korea; 2 Department of Emergency Medicine, Seoul St. Mary’s Hospital, College of Medicine, The Catholic University of Korea, 222 Banpo-daero, Seocho-gu, Seoul, 06591 Republic of Korea; 3 Department of Emergency Medicine, St. Vincent’s Hospital, College of Medicine, The Catholic University of Korea, Suwon, Gyeonggi-do Republic of Korea

**Keywords:** Attempted suicide, Older adults, Young adults

## Abstract

**Background:**

Attempted suicide remains difficult for clinicians to predict with some established risk factors. We investigate the detailed characteristics of attempted suicide especially according to age and methods of suicide attempts.

**Methods:**

A retrospective study was conducted to evaluate patients who visited the emergency department due to attempted suicide. A retrospective medical record review of all patients who presented to the emergency department (ED) of two tertiary teaching hospitals in Korea after suicide attempt between January 1, 2010, and December 31, 2017 was performed. Demographic information and detailed variables (methods and reasons of suicide attempts and variables regarding reattempts) were investigated. Total participants were classified into 3 groups according to age, young group, middle aged group and the older group, and each characteristics were compared.

**Results:**

A total of 3698 patients were enrolled in this study. Deliberate self-poisoning (DSP) was the most common method of attempted suicide (66.5%) followed by cutting (24.4%), hanging (7.9%), falling (2.6%), and drowning (1.1%). In patients who had previous suicide attempts (n = 1029, 27.8%), attempted methods were likely to be concordant with previous attempted methods. The most common reason for suicide attempts was interpersonal relationship issues followed by socio-economic reasons. Older patients (n = 412, 11.2%) were significantly different from other 2 groups (n = 3286, 88.8%) regarding gender, suicide re-attempt, occupation, alcohol co-ingestion, previous psychiatric history, and discharge outcomes (all p-values < 0.001). Especially, in older patients, use of critical method and reason of physical illness were more common.

**Conclusion:**

Our findings indicated that people who attempted suicide might have different sociodemographic and clinical factors depending on age group. Depending on age, it is necessary to apply additional suicide intervention programs in different ways.

## Background

Every 40s, a person dies by suicide somewhere in the world. Suicide kills approximately 800,000 people in a year, accounting for approximately 1.4% of all deaths worldwide [1]. A significantly high suicide rate has been reported in South Korea compared with other countries for several years [2]. The suicide rate in South Korea is the second highest among the Organization for Economic Co-Operation and Development (OECD) member countries at 25.8 deaths per 100,000 people, which is approximately 2.2 times higher than the OECD average of 11.6 deaths per 100,000 people [3]. Thus, suicide prevention is a major public health issue in South Korea. Although national approaches are being taken to prevent suicide, suicide rates are steadily increasing. In addition, the suicide rate of the older population in South Korea is the highest among the 34 OECD countries [4]. South Korea is now entering a rapidly aging society, and the older population is expected to increase to 20.8% by 2026 [5]. Therefore, the high suicide rate among older adults has become an alarming public health issue in South Korea.

Many studies have focused on suicide prevention, but it is impossible to determine the most effective suicide prevention method. However, most researchers have identified methods to prevent suicide, and targeting people who attempted suicide and helping them avoid repeated attempts are considered to be the most effective management strategies. Attempted suicide is the most powerful known risk factor for completed suicide [6–8]. According to a Swedish study, the rate of suicide among individuals in the year after a suicide attempt was approximately 100-fold higher than the corresponding suicide rate among age-and sex-matched community control individuals [9]. During the first year after a suicide attempt, the risk for completed suicide varies from 0.8 to 3.0% for men and from 0.3 to 1.9% for women [7, 9–11]. Although common risk factors for suicidal behavior have been identified, there are no factors to accurately predict who will be involved in or die from suicidal behavior [12, 13].

In the present study, we focused on the epidemiological and socio-environmental factors that lead to attempted suicide. The main aim of this study was to investigate the potential risk factors associated with attempted suicide and the characteristics of patients based on age group. Our results could help to develop and refine strategies for preventing further suicide attempts and completed suicides.

## Materials and methods

We retrospectively reviewed the medical records of attempted suicide patients who were treated at emergency departments (ED) of two sister hospitals between January 1, 2010 and December 31, 2017.

In South Korea, patient information on the cause of ED admission is immediately reported to the National Emergency Department Information System (NEDIS) records. If the cause of admission was deliberate self-harm, information on the method used for each attempted suicide was recorded as either poisoning, cutting or piercing, suffocation, hanging or choking, drowning or near drowning, using fire or heat, falling or jumping from a great height, other method, or unknown. All patients who had visited our ED after attempted suicides were included in the NEDIS records. To reduce missing data, we have supplemented as much data as possible through post management, outpatient clinic, consultation, and reviewing medical records.

We included all patients who presented with objective evidence of a suicide attempt, and we excluded patients who did not present with objective evidence of a suicide attempt. We assessed the patients’ age, gender, history of suicide attempts, time interval between past suicide attempts, occupation, living condition (with or without family), alcohol co-ingestion, previous psychiatric history, reason for the suicide attempts and outcome. Additionally, patients who had previously attempted suicide were investigated to determine the previously selected method. When two or more methods were used as suicide attempts, duplication was investigated. In addition, if two or more reasons for suicide attempts were noted, duplication was investigated. Two emergency physicians independently reviewed the medical service records, medical records and psychiatric records. Any discrepancies were arbitrated by a third investigator.

In the present study, the overall characteristics of patients who attempted suicide and the differences between young adults and the older were investigated. In addition, we investigated the time from the first suicide attempt to the re-attempt for patients who re-attempted suicide.

The distribution of patient characteristics is presented as either a percentage or the mean ± standard deviation. To compare the distribution of the characteristics between the two groups, we performed Student’s t-tests to analyze continuous variables and Chi squared tests to analyze categorical variables. All statistical analyses were performed using SPSS 16 (SPSS, Chicago, IL), and differences with a p-value < 0.05 were considered statistically significant. Especially for predicting re-attempt associated factor, logistic regression analysis was performed. all variables with a significance level of p < 0.05 in the univariate analysis were included in a multivariable logistic regression model, and the odds ratios (ORs) and 95% confidence intervals (CIs) were estimated.

The institutional review board of The Catholic University of Korea, Seoul Saint Mary’s Hospital approved the study protocols prior to data analysis. Informed consent was waived given the retrospective nature of the study.

## Results

**Fig. 1:**
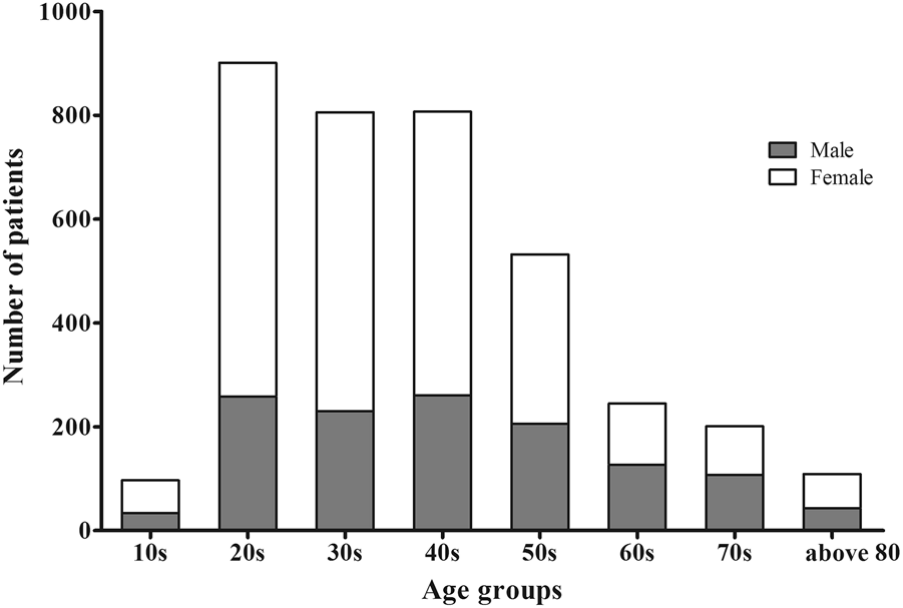
Number of patients by age group between male and female

**Fig. 2:**
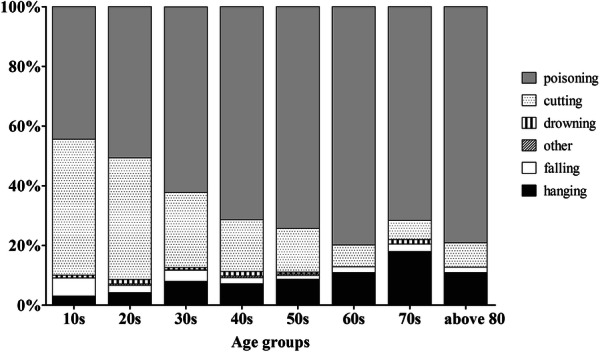
Suicide methods by age group

During the study period, 3698 attempted suicide patients were admitted to the ED, constituting 0.34% of all ED visits. The characteristics of the patients are shown in Table 1. Of these patients, 1266 (34.2%) were male and 2432 (65.8%) were female. The mean age of the patients was 42.1 ± 16.6 years. Among the patients who attempted suicide, twenties were the most. And the younger, the higher the proportion of female among those who attempted suicide, and the older, the higher the proportion of male (Fig. 1). The most common method of attempted suicide was deliberate self-poisoning (DSP) (66.5%), followed by cutting (24.4%), hanging (7.9%), falling (2.6%), and drowning (1.1%). In Fig. 2, we investigate the methods in which suicide attempts were made by age group. Cuttings account for a larger proportion of younger patients, while the proportion of DSP increases with age. In the case of older adults, the proportion of hanging is higher than that of younger people.

**Table 1:** Prevalence of attempted suicide by sociodemographic and clinical variable (n = 3698)

	n	%
Gender		
Male	1266	34.2
Female	2432	65.8
Age, mean ± SD, years	42.1 ± 16.6	
–19	97	2.6
20–29	901	24.4
30–39	806	21.8
40–49	807	21.8
50–59	532	14.4
60–69	245	6.6
70–79	201	5.4
80–	109	2.9
Re-attempt	1029	27.8
Occupation		
Unemployed	1358	36.7
Employed (including student)	1420	38.4
Housewife	920	24.9
Living condition		
No family	544	14.7
Family	3154	85.3
Alcohol co-ingestion	1554	42.0
Previous psychiatric history	1641	44.4
Suicide attempt method		
Poisoning	2461	66.5
Cutting	904	24.4
Drowning	41	1.1
Falling	96	2.6
Hanging	292	7.9
Other methods	12	0.3
Psychiatric interview	2439	66.0
In-hospital mortality	218	5.9

The study population’s reasons for suicide attempts are shown in Table 2. Interpersonal relationship issues (51.8%) were the most common reasons. Of these patients, the most common type of interpersonal relationship issue was couple conflict (32.3%). Especially, there were significantly differences between the older people and the others in couple conflict. And financial stress was significantly highest in middle aged group (p < 0.001). While that, the physical illness is the most common in the older group significantly (p < 0.001). Among social issues, financial stress was the most common reason for suicide attempts.

**Table 2:** Reasons for suicide attempts

Reasons	Total (N = 3698)	Older (N = 412)	Middle aged (N = 1777)	Young (N = 1509)	*p*
Interpersonal relationship issues					
a. Couple conflicts	1193 (32.3)	55 (13.3)	621 (34.9)	517 (34.3)	< 0.001
b. Family conflicts	548 (14.8)	57 (13.8)	268 (15.1)	223 (14.8)	0.813
c. Others	175 (4.7)	9 (2.2)	77 (4.3)	89 (5.9)	0.004
Social issues					
a. Job loss	87 (2.4)	8 (1.9)	33 (1.9)	46 (3.0)	0.068
b. Financial stress	499 (13.5)	35 (8.5)	323 (18.2)	141 (9.3)	< 0.001
c. Work stress	170 (4.6)	0 (0.0)	67 (3.8)	103 (6.8)	< 0.001
d. Legal academic stress	42 (1.1)	3 (0.7)	27 (1.5)	12 (0.8)	0.106
Psychiatric illness					
a. Mood disorder: depressive, bipolar	356 (9.6)	39 (9.5)	156 (8.8)	161 (10.7)	0.186
b. Adjustment disorder	3 (0.1)	0 (0.0)	2 (0.1)	1 (0.1)	0.744
c. Schizophrenia, other psychotic dis	119 (3.2)	7 (1.7)	55 (3.1)	57 (3.8)	0.098
d. Anxiety disorder	16 (0.4)	1 (0.2)	9 (0.5)	6 (0.4)	0.736
e. Panic disorder	14 (0.4)	0 (0.0)	5 (0.3)	9 (0.6)	0.142
f. Substance-related	83 (2.2)	10 (2.4)	54 (3.0)	19 (1.3)	0.003
g. Personality disorder	31 (0.8)	1 (0.2)	6 (0.3)	24 (1.6)	< 0.001
Physical illness					
a. Oneself	275 (7.4)	149 (36.2)	107 (6.0)	19 (1.3)	< 0.001
b. Others	68 (1.8)	22 (5.3)	34 (1.9)	12 (0.8)	< 0.001
Death of acquaintance					
a. Spouse	28 (0.8)	17 (4.1)	9 (0.5)	2 (0.1)	< 0.001
b. Family	54 (1.5)	4 (1.0)	23 (1.3)	27 (1.8)	0.339
c. Others	18 (0.5)	0 (0.0)	8 (0.5)	10 (0.7)	0.220

Parentheses indicate percentage

**Fig. 3:**
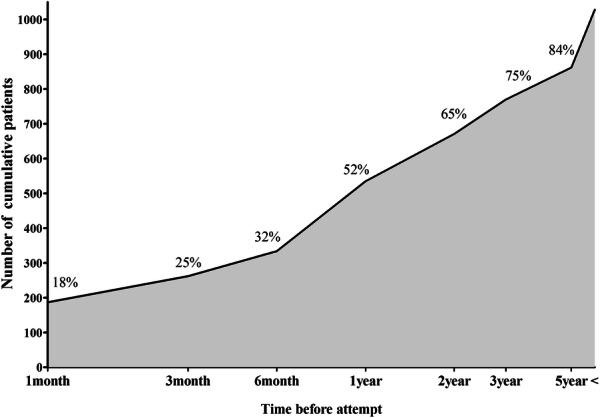
The incidence of repeated suicide attempt time

Among the patients with repeated suicide attempt (n = 1029, 27.8%), the incidence of repeated suicide attempt within one, two to three, four to six, and seven to twelve months was 18%, 7%, 7% and 20%, respectively. Of the patients who visited the ED due to suicide attempts, 52% of the patients repeated suicide attempt within one year (Fig. 3).

The previous suicide attempt methods used by the patients with repeated suicide attempts are shown in Table 3. The common previous suicide attempt method was DSP (48.4%) and cutting (40.1%). Attempted suicide methods were likely to use previous attempted methods. In the DSP group (n = 659), the most common previously used method was DSP (n= 502, 62.8%), and the second most common was cutting (n = 214, 26.8%). In the cutting group (n = 306), the most common previously used method was cutting (n = 249, 72.8%), and the second most common was DSP (n = 64, 18.7%). But in critical methods, such as drowning, falling and hanging, Cutting and DSP have been attempt more before than using the same previous method.

**Table 3:** The previous suicide attempt methods used by the patients with repeated suicide attempts

Re-attempt method (N = 1029)	Poisoning	Cutting	Drowning	Falling	Hanging	Others
	N = 659	N = 306	N = 5	N = 19	N = 36	N = 4
Previous methods						
Poisoning (n = 593, 48.4%)	502 (62.8)	64 (18.7)	1 (16.7)	5 (19.2)	20 (41.7)	1 (25.0)
Cutting (n = 492, 40.1%)	214 (26.8)	249 (72.8)	4 (66.7)	12 (46.2)	12 (25.0)	1 (25.0)
Drowning (n = 12, 1.0%)	8 (1.0)	3 (0.9)	0 (0.0)	1 (3.8)	0 (0.0)	0 (0.0)
Falling (n = 37, 3.0%)	20 (2.5)	9 (2.6)	1 (16.7)	4 (15.4)	3 (6.3)	0 (0.0)
Hanging (n = 77, 6.3%)	48 (6.0)	13 (3.8)	0 (0.0)	4 (15.4)	12 (25.0)	0 (0.0)
Other (n = 15, 1.2%)	8 (1.0)	4 (1.2)	0 (0.0)	0 (0.0)	1 (2.1)	2 (50.0)

Parentheses indicate percentage

Table 4 shows the results of comparing the factors related to suicide attempts by distinguishing young, middle aged adults from older people. Gender, suicide re-attempt, occupation, alcohol co-ingestion, previous psychiatric history, and methods of suicide attempt were significantly different among the groups (p < 0.001). Hanging was the most common suicide method in the older group (14.8% vs. 8.0%, 5.9%, respectively), while cutting was the most in young people. And the hospital death rate was significantly higher in older patients than the young and middle aged group (p < 0.001).

**Table 4:** Comparison of characteristics between young, middle aged and older people groups. (N = 3698)

	Older	Middle aged	Young	*p*
	n = 412	n = 1777	n = 1509	
Gender				< 0.001
Male	207 (50.2)	626 (35.2)	433 (28.7)	
Female	205 (49.8)	1151 (64.8)	1076 (71.3)	
Age, mean SD	75.2 ± 6.8	47.5 ± 7.5	26.7 ± 5.0	< 0.001
Re-attempt	55 (13.3)	433 (24.4)	541 (35.9)	< 0.001
Occupation				< 0.001
Unemployed	329 (79.9)	411 (23.1)	618 (41.0)	
Employed(including student)	34 (8.3)	669 (37.6)	717 (47.5)	
Housewife	49 (11.9)	697 (39.2)	174 (11.5)	
Living condition				0.860
No family	57 (13.8)	262 (14.7)	225 (14.9)	
Family	355 (86.2)	1515 (85.3)	1284 (85.1)	
Alcohol co-ingestion	88 (21.4)	819 (46.1)	647 (42.9)	< 0.001
Previous psychiatric history	130 (31.6)	778 (43.8)	733 (48.6)	< 0.001
Suicide attempt method				
Poisoning	314 (76.2)	1312 (73.8)	835 (55.3)	< 0.001
Cutting	30 (7.3)	293 (16.5)	581 (38.5)	< 0.001
Drowning	3 (0.7)	21 (1.2)	17 (1.1)	0.728
Falling	8 (1.9)	36 (2.0)	52 (3.4)	0.026
Hanging	61 (14.8)	142 (8.0)	89 (5.9)	< 0.001
Other methods	0 (0.0)	8 (0.5)	4 (0.3)	0.305
Psychiatric interview	271 (65.8)	1199 (67.5)	969 (64.2)	0.145
In-hospital mortality	73 (17.7)	93 (5.2)	52 (3.4)	< 0.001

Parentheses indicate percentage

In multivariate regression analysis revealed a significant association between re-attempts and age groups (aOR: 3.04 and 1.77, young and middle aged group compared with older group, respectively) and a previous psychiatric history (OR: 5.65, 95% CI 4.79–6.66, p < 0.001) (Table 5).

**Table 5:** Univariate and multivariate logistic regression analysis for predicting re-attempt events

	Univariate		Multivariate	
	OR (95% CI)	p	aOR (95% CI)	p
Female	1.53 (1.31–1.80)	< 0.001	1.15 (0.97–1.37)	0.106
Age group				
Young people	3.63 (2.68–4.91)	< 0.001	3.04 (2.21–4.19)	< 0.001
Middle aged people	2.09 (1.54–2.83)	< 0.001	1.77 (1.28–2.44)	< 0.001
Older people	Reference			
Unemployed	1.02 (0.88–1.18)	0.754		
Living without family	1.17 (0.96–1.43)	0.106		
Previous psychiatric history	5.89 (5.01–6.92)	< 0.001	5.65 (4.79–6.66)	< 0.001
Suicide attempt method				
Poisoning	0.92 (0.75–1.12)	0.423		
Cutting	0.92 (0.74–1.14)	0.456		
Drowning	0.23 (0.30–1.82)	0.166		
Falling	1.41 (0.71–2.78)	0.321		
Hanging	1.03 (0.63–1.71)	0.883		

*OR* odds ratio, *CI* confidence interval, *aOR* adjusted odds ratio

## Discussion

In the present study, we sought to summarize detailed characteristics of attempted suicide according to age and methods of suicide attempts. The general findings of the patients who presented to our ED after attempted suicide were as follows: attempted suicide was more prevalent in females (especially in young adults), the most common method of attempted suicide was deliberate self-poisoning, repeated suicide attempts were noted in 27.8% (of which 52% were within one year), and interpersonal relationship issues (51.8%) were the most common reasons (physical illness was most common reason in older individuals).

Regarding gender differences, women attempted suicide more frequently than men. However, the suicide attempt rates do not differ among people who are 65 years or older; this result consistent with previous findings [14]. Regarding the methods of suicide attempt, DSP was the most common in all ages as described in previous studies [15, 16]. The higher the age, the higher the rate of attempting suicide with DSP. In contrast, the younger the age, the more often the suicide attempt was made by cutting.

Among patients presenting with suicide attempts, the incidence of a previous suicide attempt was 27.8% (Table 1), and approximately 14.5% of patients who attempt suicide make a second attempt during the following year. These findings are similar to the findings of a previous report [17]. Respectively, in the case of poisoning, 62.8% and in the case of cutting, 72.8% attempted suicide attempt by the same method. Only 5.8% of patients with a previous suicide attempt that involved cutting re-attempted suicide by hanging, drowning, and falling. However, these methods are less common but are classified as high lethality methods and have a high risk of completed suicide [18, 19]. In addition, a large proportion of patients who attempted suicide by cutting are discharged without undergoing a psychiatric interview because they do not wait for a psychiatric consultation after their wounds have been treated [20]. According to a report by Hickey et al., patients who have not received a psychiatric assessment may be at a greater risk of further attempted suicide and completed suicide compared with patients who undergo psychiatric assessment [21]. Therefore, a multidisciplinary team that includes a psychiatrist should assess patients who visit an ED after an attempted suicide by cutting.

Interpersonal relationship issues, especially couple conflicts were the most common cause of suicide attempt in young and middle aged people, while physical illness was cited as the most common reason in older people. And in meddle aged people the financial stress was revealed the more reason of suicide compared the other groups. This shows that different age groups have different reasons for suicide attempts, so it is thought that access to patients who have visited the hospital for suicide attempt be different. For example, it would be helpful for middle aged people to come up with measures for socioeconomic support, while for older people, policies such as continuing mental support through psychiatric connections will be needed to prevent depression in patients who are physically ill. Issues surrounding physical illnesses, such as physical symptom burden, functional disability, social effects of a handicap, and receiving a diagnosis of critical illness, have been repeatedly reported to be associated with suicidal thoughts and behaviors [22–24]. Physical illness has been assumed to be a strong risk factor for older adults suicide in previous studies, and its association with suicide ideation and attempts remained significant even after correction for mental illness as its likely consequence [25–27]. Although psychological aspects are important in the treatment of suicide patients, more attention should be paid to the underlying physical causes of attempted suicide especially in older adults. To prevent suicide attempts in the older population, we must actively investigate suicidal thoughts and depression in patients with physical illnesses in the primary care settings.

Comparing young adults and older patients, differences in gender, suicide re-attempt, alcohol co-ingestion, previous psychiatric history, methods of suicide attempts and outcomes are noted. Regarding outcomes, the mortality rate of older patients after admission was significantly higher than that of young adults. This finding might be partially explained not only by their higher physical vulnerability but also by their higher suicide intent. According to a report by Kato et al., older patients are less likely to be found and rescued compared with young adults, possibly leading to serious medical conditions and resulting in a higher risk of death and longer stay in the hospital, particularly in the ICU [28]. In addition, our results show that significantly more patients in the older group had attempted suicide by high lethality methods, such as hanging. Therefore, to prevent suicide attempts or suicide of the older adults, it is important for the primary care physician to identify the suicidal ideation, and psychiatric treatment should be immediately provided. As the results of the screening program for depression in the older adults showed that suicide was effectively reduced, a social system that can recognize the risk factors of suicide attempt or suicide seems to be important [29].

In this study, the previous psychiatric history was the most powerful risk factor for re-attempts. And the younger the patients compared to the older patients, the more likely they were to re-attempts. However, the previous suicide attempt method was not associated with re-attempts. These results will help us to find an adequate suicide prevention strategy.

There are several limitations to our study. First, the data are restricted to two tertiary hospitals in Seoul and Gyeongido, South Korea. This region may not be representative of the entire country. Furthermore, patients who re-attempt suicide may have visited another hospital for a previous attempt. Multicenter trials are needed to identify more generalizable risk factors and acquire more accurate data. Second, several known risk factors associated with attempted suicide could not be obtained; certain factors could be more specific. Alcohol dependence, low socioeconomic status, low educational level, unmarried status, traumatic or abusive experiences during childhood, and emotions (i.e., intense feelings of despair, the loss of one’s sense of social relevance and the meaning of one’s existence) are all risk factors for suicide attempt and re-attempts [30–32]. And this study was cross-sectional analysis, so does not allow drawing causal conclusions. These factors should be evaluated in further studies.

Nevertheless, this study included large samples considering the population of the city and the study period, so the data are expected to reveal some degree of suicide attempts in the region. Prospective cohort design studies are needed to minimize participant loss and study various factors.

## Conclusion

This study attempted to provide information on the characteristics of adult suicide attempters in South Korea. Knowledge of the risk factors associated with attempted suicide can help identify the populations who are exposed to injury or death by suicide attempts. Findings from this study indicate that people who attempt suicide may have different sociodemographic and clinical factors depending on age group. In particular, the reason and method for attempting suicide in the older people were quite different compared with young or middle aged people. Thus, a tailored suicide intervention program for each age group is needed. In future studies, it is necessary to examine the effects of interventions that target these risk factors to reduce attempted suicide and suicide.

## Data Availability

The datasets used and/or analysed during the current study are available from the corresponding author on reasonable request.

## Abbreviations

ED: Emergency department
DSP: Deliberate self-poisoning
OECD: Organization for Economic Co-Operation and Development
NEDIS: National Emergency Department Information System
